# Elevated GDF15 in ocular fluids of patients with proliferative diabetic retinopathy

**DOI:** 10.3389/fendo.2026.1882124

**Published:** 2026-07-16

**Authors:** Zelu Wang, Xiaoxia Li, Liang Fu, Xuemei Zhu, Yaoyao Sun, Yuou Yao, Mingwei Zhao, Mingwu Li, Enzhong Jin, Wenzhen Yu

**Affiliations:** 1 Department of Ophthalmology, Peking University People’s Hospital, Beijing, China; 2 Key Laboratory of Ocular Disease and Optometry Science, Peking University People’s Hospital, Beijing, China

**Keywords:** aqueous humor, enzyme-linked immunosorbent assay, growth differentiation factor 15 (GDF15), proliferative diabetic retinopathy (PDR), vitreous humor

## Abstract

**Background/objective:**

Growth differentiation factor 15 (GDF15) is a multifunctional stress-responsive cytokine widely involved in inflammation, oxidative stress, and microvascular injury. Its expression profile in the local intraocular microenvironment of patients with proliferative diabetic retinopathy (PDR) remains unclear. This study aimed to detect GDF15 levels in aqueous humor (AH) and vitreous humor (VH) of PDR patients, analyze its differential expression, and provide preliminary evidence for understanding the intraocular expression pattern of GDF15 in PDR.

**Methods:**

GDF15 levels were quantified by enzyme-linked immunosorbent assay (ELISA) in AH and VH samples from PDR patients and compared with those in AH from age-related cataract patients and VH from patients with macular hole or epiretinal membrane.

**Results:**

GDF15 levels in AH of PDR patients were significantly higher than those in cataract controls; GDF15 levels in VH of PDR patients were markedly higher than those in macular hole/epiretinal membrane controls. An exploratory comparison between AH and VH samples from independent PDR patient cohorts showed higher GDF15 levels in VH than in AH.

**Conclusion:**

GDF15 expression is significantly upregulated in AH and VH of PDR patients, with a potential difference between ocular fluid compartments, suggesting an association between intraocular GDF15 upregulation and PDR. These findings provide preliminary evidence for further investigation of GDF15-related inflammatory and stress responses in the local intraocular microenvironment of PDR.

## 1 Introduction

GDF15 is a secreted ligand of the transforming growth factor-β (TGF-β) superfamily and a pleiotropic cytokine (1, 2). Under physiological conditions, GDF15 is expressed at low levels in most organs, but is significantly upregulated under pathological conditions such as tissue hypoxia, inflammation, oxidative stress, and cell injury (2). It is involved in regulating various core cellular processes including apoptosis, angiogenesis, and inflammation (1). Meanwhile, it has been widely studied as a stress-related molecule in cardiovascular diseases, tumors, and metabolic diseases, and has been researched in cardiovascular diseases, tumors, and metabolic diseases (1, 3).

Diabetic retinopathy is the most common chronic microvascular complication of diabetes, and its pathogenesis is closely related to inflammation, oxidative stress, and retinal microvascular damage (4, 5). GDF15 may be associated with retinal inflammatory responses (6), and its plasma level is positively correlated with the occurrence and development of diabetic retinopathy, increasing significantly with the severity of the disease (6). However, studies on the specific expression level, distribution characteristics of GDF15 in the local intraocular microenvironment of diabetic patients and its internal relationship with diabetic ocular diseases are still scarce, no clear conclusion has been formed, and its mechanism of action in the local eye has not been fully elucidated.

This study aimed to detect the specific content of GDF15 protein in VH and AH samples of diabetic patients, provide preliminary clinical evidence of intraocular GDF15 expression in PDR, and furnish preliminary clinical evidence for further investigating the local expression characteristics of GDF15 in the intraocular microenvironment of PDR.

## 2 Methods

### 2.1 Patient recruitment

A total of 15 AH samples and 14 VH samples were collected from patients with PDR. Meanwhile, control groups were recruited, including 10 AH samples from cataract patients and 9 VH samples from patients with macular hole or epiretinal membrane. All subjects were from Peking University People’s Hospital. Written informed consent was obtained from all participants, and corresponding AH or VH samples were collected according to research requirements. All study procedures were performed in accordance with the ethical standards laid down in the 1964 Declaration of Helsinki and its subsequent amendments. This study protocol was approved by the Ethics Committee of Peking University People’s Hospital (No.: 2022PHB413-001). Baseline characteristics of participants are shown in Result 3.1.

### 2.2 Diagnosis and exclusion criteria

This study adopted the internationally recognized *International Clinical Diabetic Retinopathy Disease Severity Scale (ICDR)* for diabetic retinopathy (DR) (7), patients with PDR characterized by retinal neovascularization (including disc neovascularization and retinal neovascularization), accompanied by complications such as vitreous hemorrhage and tractional retinal detachment, were enrolled in this study. All PDR patients were definitely staged by fundus photography, optical coherence tomography (OCT), and fundus fluorescein angiography (FFA). Control cataract patients had age-related cataract without retinopathy or other organic ocular diseases; patients with macular hole or epiretinal membrane were diagnosed by fundus examination and OCT, without optic neuropathy or other severe systemic diseases. All subjects were excluded if they: I had severe systemic diseases (such as severe cardiovascular and cerebrovascular diseases, hepatic and renal failure, malignant tumors, autoimmune diseases, etc.); II had ocular infections, uveitis, glaucoma or other inflammatory or structural ocular diseases; III had history of previous intraocular surgery; IV had contraindications to sample collection.

### 2.3 Sample collection and processing

AH samples were collected by anterior chamber paracentesis before cataract surgery or intravitreal injection, with a volume of 100 μL per sample; VH samples were collected during vitrectomy, with a volume of 200–300 μL per sample. After collection, samples were immediately transferred to sterile EP tubes, centrifuged at 1000 r/min for 3 min. The supernatant was aliquoted and quickly stored in a -80 °C ultra-low temperature freezer to avoid repeated freeze-thaw cycles and ensure sample activity and detection accuracy. All samples were collected during surgery under strict aseptic conditions to prevent contamination.

### 2.4 Detection and analytical methods

GDF15 protein levels in all AH and VH samples were detected by enzyme-linked immunosorbent assay (ELISA) in strict accordance with the kit instructions. The GDF15 ELISA kit was purchased from Proteintech (Cat. KE00108); the microplate reader used was Bio-Rad iMark 22440. Samples were thawed slowly at room temperature, and procedures including sample dilution, loading, incubation, washing, secondary antibody incubation, re-washing, color development and reaction termination were performed sequentially. Blank control wells, standard wells and sample duplicate wells were set to eliminate experimental interference and ensure the repeatability of detection results. All experimental data were organized, analyzed and plotted using GraphPad Prism 10.1.2 software. Outliers were excluded and the mean OD value of each sample duplicate was calculated. Standard curves were drawn based on OD values of standards. Normally distributed data with unequal variances, Welch’s corrected independent samples t-test was applied; non-normally distributed data were analyzed using the Mann–Whitney U test. For non-normal multi-group data, the Kruskal–Wallis test was conducted first, followed by Dunn’s multiple comparison test for pairwise comparisons. Spearman’s correlation analysis was adopted to evaluate the correlations between confounding factors and outcome indicators.

## 3 Results

### 3.1 Baseline characteristics

Baseline characteristics of enrolled patients are shown in **Table 1** and **Table 2**.

**Table 1:** Baseline characteristics and GDF15 levels of the enrolled subjects.

Characteristics	PDR AH (n=15)	Cataract AH (n=10)	P value	PDR VH (n=14)	MH/ERM VH (n=9)	P value
Age, years	60.33 ± 9.49	67.60 ± 6.24	0.031	50.71 ± 14.54	65.67 ± 6.96	0.004
Female sex, n (%)	6/15 (40.0%)	7/10 (70.0%)	0.226	5/14 (35.7%)	8/9 (88.9%)	0.029
Fasting plasma glucose, mmol/L	11.11 ± 4.72	6.05 ± 1.35	—	8.44 ± 3.24	4.89 ± 0.58	—
HbA1c, %	9.20 ± 2.20	NA	—	9.15 ± 1.77	NA	—
Duration of diabetes, years	25.00 ± 7.07	NA	—	11.33 ± 6.31	NA	—
Hypertension, n/N (%)	3/6 (50.0%)	4/10 (40.0%)	—	9/13 (69.2%)	2/9 (22.2%)	—
Dyslipidemia, n/N (%)	3/9 (33.3%)	4/10 (40.0%)	—	3/14 (21.4%)	2/9 (22.2%)	—
Prior PRP, n/N (%)	NA	NA	—	7/13 (53.8%)	0/9 (0.0%)	—
Prior intravitreal injection, n/N (%)	NA	NA	—	8/13 (61.5%)	0/9 (0.0%)	—
GDF15, ng/mL	16.62 ± 8.89	4.13 ± 2.13	<0.001	46.95 ± 32.59	12.13 ± 12.37	<0.001

(Values are presented as mean ± standard deviation or n/N (%). Statistical comparisons were performed for age, sex distribution, and GDF15 levels between the corresponding PDR and control groups. Other clinical variables are presented descriptively because of incomplete clinical records and intrinsic differences between diabetic and non-diabetic control subjects. Diabetes-related variables were not applicable in non-diabetic controls).

**Table 2:** Exploratory correlation analysis between intraocular GDF15 levels and available clinical variables in PDR patients.

Sample type	Variable	Recorded n	Analysis n	Spearman r	P value	Interpretation
AH	FPG	9	9	-0.400	0.286	NS
	HbA1c	6	6	-0.886	0.019	Significant
	Hypertension	6	6	0.683	0.135	NS
	Dyslipidemia	9	9	0.639	0.064	NS
VH	FPG	14	14	-0.248	0.392	NS
	HbA1c	4	4	-0.200	0.800	NS
	Duration of diabetes	6	6	0.116	0.827	NS
	Hypertension	13	13	0.802	<0.001	Significant
	Dyslipidemia	14	14	0.022	0.942	NS
	Prior PRP	13	13	-0.165	0.590	NS
	Prior intravitreal injection	13	13	-0.296	0.326	NS

(Spearman correlation analysis was performed to evaluate the relationship between intraocular GDF15 levels and available clinical variables in PDR patients. Only cases with available records for each variable were included in the corresponding analysis. The results provided for reference purposes only because of the limited sample size and incomplete clinical information.).

Compared with cataract AH controls, the PDR AH group was younger, while sex distribution showed no significant difference. Compared with MH/ERM VH controls, the PDR VH group was also younger, and the proportion of females was lower.

Clinical variables, including Fasting Plasma Glucose (FPG), Glycated Hemoglobin (HbA1c), duration of diabetes, hypertension, dyslipidemia, prior PRP, and intravitreal injection history, were summarized in **Table 1**. Due to incomplete records and inherent differences between diabetic and non-diabetic controls, some variables were presented descriptively.

Exploratory correlation analyses were performed in PDR patients with available data (**Table 2**). In the AH cohort, HbA1c was significantly associated with GDF15, whereas FPG, hypertension, and dyslipidemia were not. In the VH cohort, hypertension was significantly associated with GDF15, while FPG, HbA1c, diabetes duration, dyslipidemia, prior PRP, and intravitreal injection history showed no significant correlations.

### 3.2 Comparisons and statistical differences

**Figure 1:**
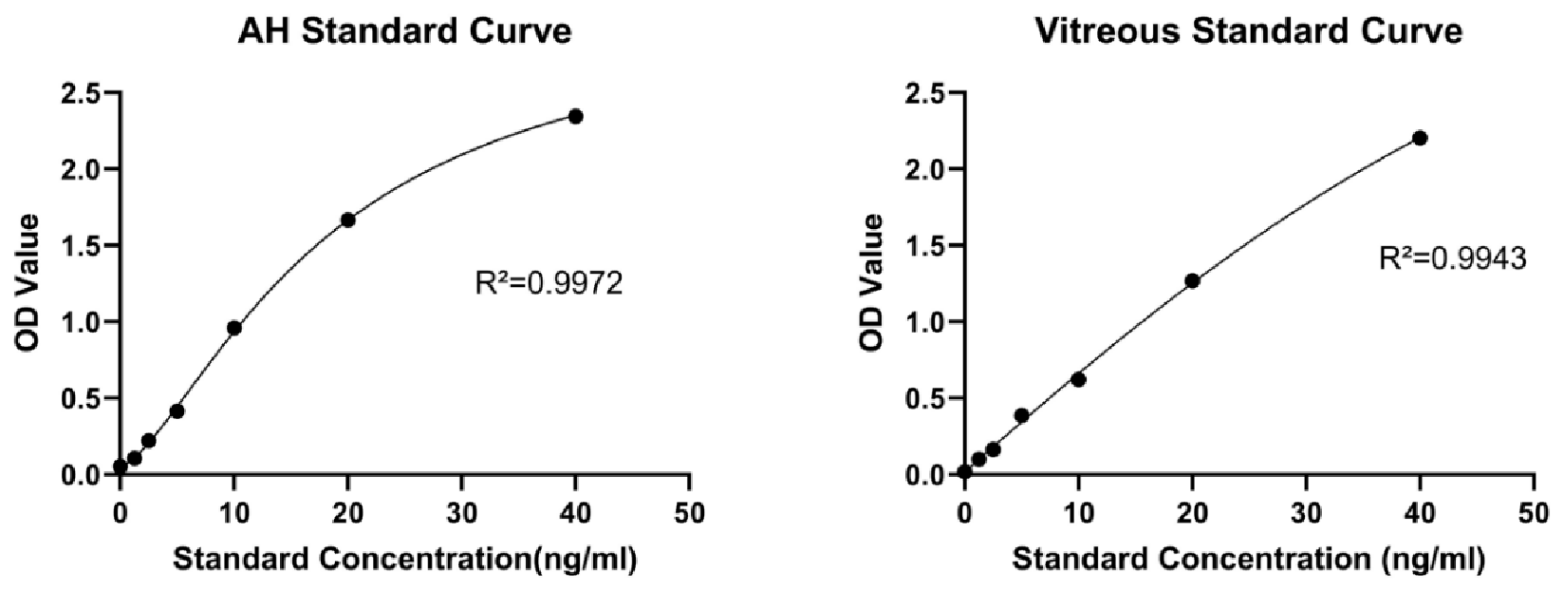
Standard curves of aqueous humor and vitreous humor specimens.

**Figure 2:**
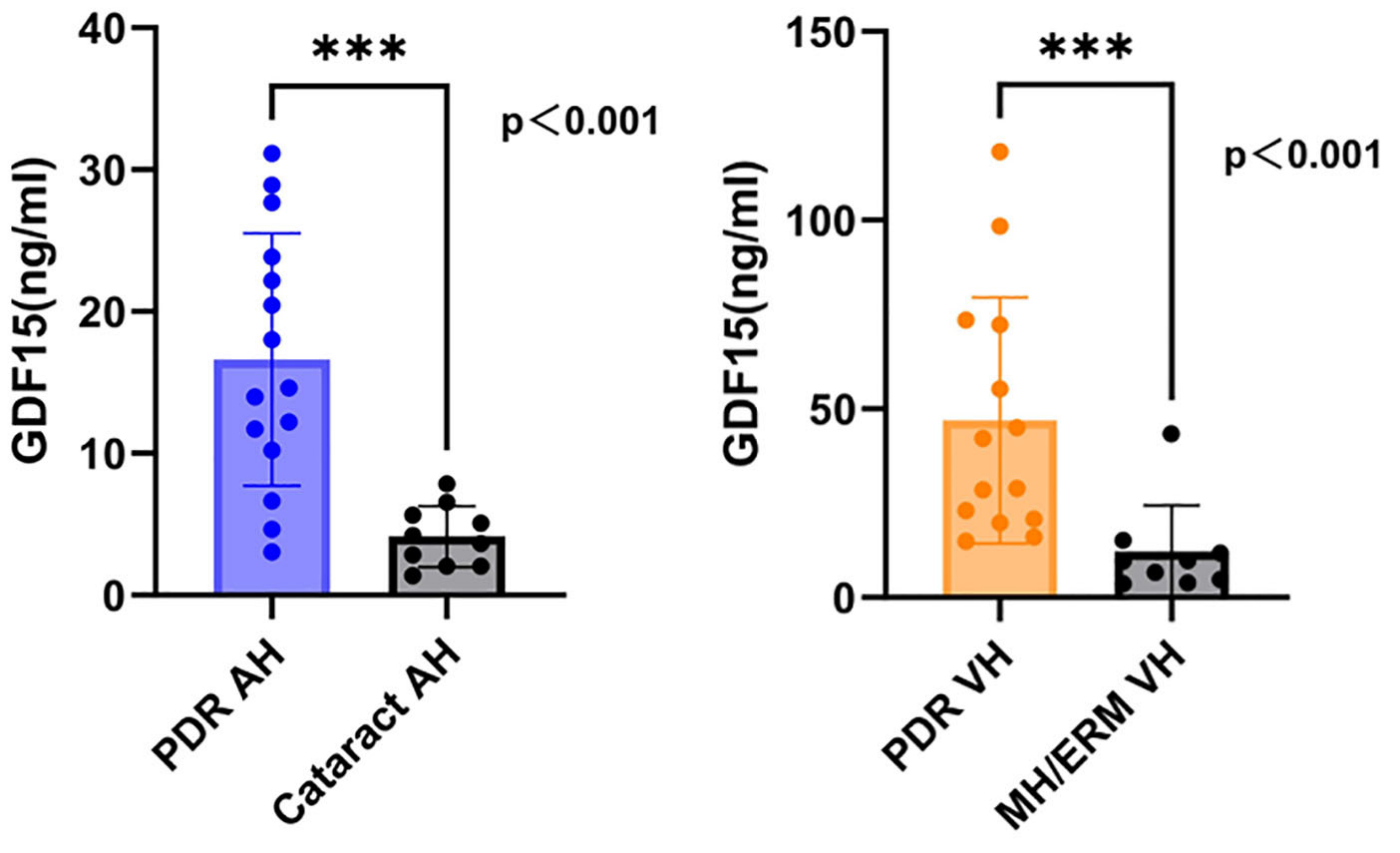
Comparison of GDF15 levels in aqueous humor and vitreous humor specimens among different groups. *** indicates p < 0.001.

The coefficient of determination (R²) of the standard curve was 0.9972 for AH samples and 0.9943 for VH samples. Both curves showed excellent linearity (**Figure 1**), meeting the quality control requirements of the kit instructions and providing a reliable basis for accurate quantification of subsequent sample concentrations. Compared with AH from age-related cataract controls, GDF15 protein concentration in AH of PDR patients was significantly increased (p<0.001, Welch t-test). Compared with VH from macular hole/epiretinal membrane controls, GDF15 concentration in VH of PDR patients was significantly increased (p<0.001, Mann–Whitney U) (**Figure 2**).

As AH and VH samples were not obtained as paired samples from the same patients, the comparison between AH and VH GDF15 levels was performed as an exploratory inter-sample analysis. The results showed that GDF15 protein concentration was higher in VH than in AH among PDR samples. Although this finding should be interpreted cautiously because of the non-paired sample design and limited sample size, it may suggest a potential compartment-related distribution pattern of GDF15 in the intraocular microenvironment of PDR and provides useful preliminary evidence for future paired-sample studies.

**Figure 3:**
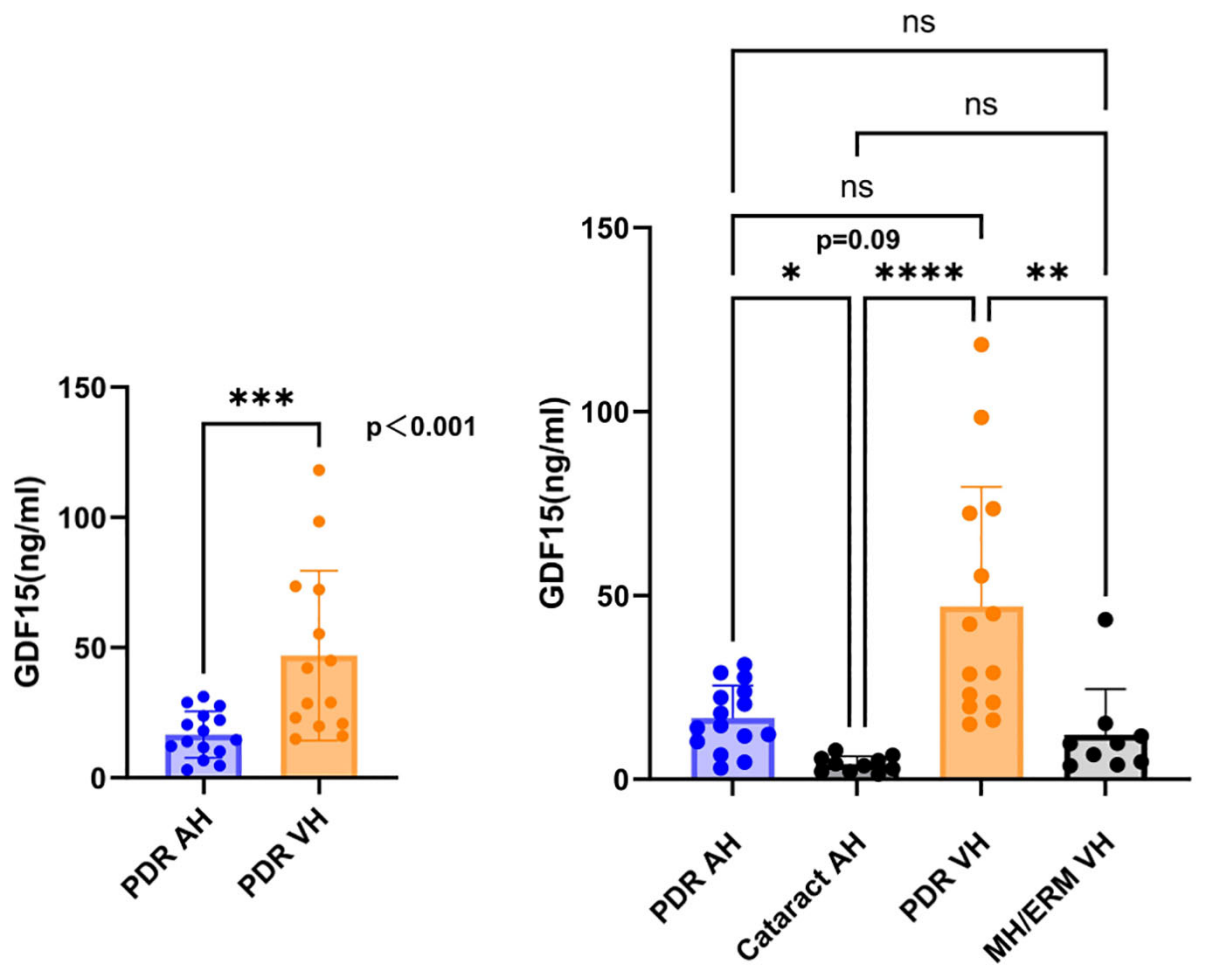
Exploratory comparison of GDF15 levels between AH and VH in PDR and control groups from independent PDR patient cohorts. * indicates p < 0.05, ** indicates p < 0.01, *** indicates p < 0.001, and **** indicates p < 0.0001. “ns” indicates not significant.

Although analysis of AH and VH in different PDR patients showed insufficient statistical significance (p=0.09, Kruskal–Wallis and Dunn’s test) in multi-group analysis, separate comparison revealed that GDF15 protein concentration in VH was significantly higher than that in AH (p<0.001, Mann–Whitney U). There was no significant difference in GDF15 levels between control groups (cataract AH vs macular disease VH) (**Figure 3**).

## 4 Discussion

GDF15 has been confirmed to be significantly upregulated under various pathological conditions such as metabolic disorders, chronic inflammation, tissue ischemia and injury repair, and is regarded as a molecule reflecting systemic stress burden and disease progression (1, 8). Its canonical receptor GFRAL is mainly highly expressed in the area postrema and nucleus tractus solitarius of the central nervous system, with low expression in peripheral tissues and local ocular microenvironment (1, 9). GDF15 exhibits typical dual functional characteristics. In terms of protection, as an adaptive stress factor, it is associated with maintaining cell homeostasis, inhibiting apoptosis and exerting neuroprotective effects (2, 10). In terms of pathology, GDF15 is related to chronic inflammation, tissue fibrosis and endothelial dysfunction in cardiovascular and metabolic diseases, also related to inflammatory amplification, extracellular matrix deposition and vascular remodeling, and can be used to reflect disease severity and poor prognosis (1, 2, 11).

At present, GDF15 has been proven to exert potential functions in a variety of ocular diseases. Significantly elevated aqueous humor GDF15 levels have been detected in glaucoma patients and are correlated with disease severity (12–14). Upregulated retinal GDF15 protein expression has also been observed in mouse and rat optic nerve crush models (15). In addition, GDF15 is associated with the functional regulation of microglia in autoimmune uveitis models (16). These findings collectively indicate that GDF15 plays an role in ocular inflammation and injury processes, while clinical studies on the expression characteristics of GDF15 in DR, especially PDR, which is also predominantly characterized by inflammatory responses and oxidative stress, are still scarce, particularly in aqueous humor and vitreous humor.

Previous studies mostly focused on confirming the elevation of classic inflammatory and angiogenic factors such as VEGF, IL-6, IL-8 and IL-17 in ocular fluids, suggesting a persistent imbalance of inflammation-angiogenesis in the intraocular microenvironment (17–19). VEGF is a central pro-angiogenic cytokine in PDR and plays a key role in retinal neovascularization and vascular permeability. Although VEGF levels were not measured in the present study due to limited sample availability, previous evidence suggests a potential biological relationship between GDF15 and VEGF-related angiogenic pathways. GDF15 has been reported to promote angiogenesis under hypoxic conditions through modulation of the p53/HIF-1α signaling pathway (20), and other studies have suggested that GDF15-related angiogenic responses may involve VEGF-associated signaling (21). Therefore, combined detection of GDF15 and VEGF in ocular fluids may provide further insight into the inflammatory-angiogenic network of PDR and should be considered in future prospective studies.

Meanwhile, studies have shown that complement fragments in AH and VH of PDR patients have a good correlation (19, 22), supporting that AH can be used as an alternative detection sample for intraocular microenvironment under certain conditions. Both AH and VH are widely used intraocular fluids for studying retinal diseases. In PDR, breakdown of the blood-retinal and blood-aqueous barriers lead to intraocular accumulation of cytokines, making ocular fluids reliable indicators of local retinal pathology. Vitreous VEGF has been shown to be significantly elevated in PDR and correlates with disease severity, supporting its role as a direct marker of retinal activity. In addition, aqueous humor VEGF is also increased in PDR and shows correlation with vitreous levels and disease status, indicating that AH can serve as a minimally invasive surrogate when VH sampling is not feasible (23, 24). These findings support the biological relevance of both AH and VH in reflecting intraocular inflammatory and angiogenic changes in PDR.

The results of this study showed that GDF15 levels in AH and VH of PDR patients were significantly higher than those in corresponding controls, and there was no significant difference between controls. This is highly consistent with the biological characteristics of GDF15 as a stress-inflammatory signaling molecule, and also consistent with previous research conclusions that inflammatory mediators in PDR eyes are generally upregulated. During the analysis of baseline characteristics in the present study, we observed demographic differences in age and sex distribution between the PDR group and the control group, which were more pronounced in the vitreous subgroup. These discrepancies were partly attributable to the limited sample size and the inherent difficulty in obtaining age- and sex-matched intraocular fluid samples from non-diabetic surgical control subjects in real-world clinical settings, particularly vitreous samples. Therefore, potential bias arising from demographic imbalance should be considered when interpreting the findings. GDF15 is a stress-responsive molecule, and its expression may be influenced by metabolic status, systemic vascular comorbidities, and prior ocular treatments. In the present exploratory analysis, most available clinical variables, including fasting plasma glucose, dyslipidemia, prior pan-retinal photocoagulation, and previous intravitreal injection history, were not significantly associated with intraocular GDF15 levels. However, HbA1c in the aqueous humor subgroup and hypertension in the vitreous subgroup showed significant associations with GDF15 levels. These findings suggest that metabolic control and systemic vascular conditions may, to some extent, influence intraocular GDF15 expression. Nevertheless, these results should be interpreted with caution due to the limited sample size and incomplete clinical data. In summary, GDF15 levels were concurrently elevated in both aqueous humor and vitreous humor of patients with PDR, supporting an association between intraocular upregulation of GDF15 and PDR. However, the potential confounding effects of demographic and systemic factors cannot be fully excluded. Future prospective studies with larger sample sizes, better-matched control groups, and more comprehensive clinical data are warranted to further determine whether elevated intraocular GDF15 is independently associated with disease severity and activity in PDR.

AH and VH samples from PDR patients were also analyzed. Because these samples were obtained from different patient cohorts rather than paired samples from the same individuals, the comparison between AH and VH represents an exploratory inter-group analysis rather than an intra-individual paired comparison. Although not based on paired design, the observation that GDF15 levels were higher in VH than in AH remains possibly meaningful. This finding may suggest a potential compartment-related distribution pattern of GDF15 within the intraocular microenvironment of PDR and is consistent with the pathological characteristics dominated by ischemic injury in the posterior segment of the retina. However, this result should be interpreted as hypothesis-generating rather than mechanistic evidence. The lack of statistical significance may be attributable to limited sample size and inter-individual variability. Nevertheless, this trend provides valuable preliminary evidence to support future well-designed paired-sample studies aimed at further clarifying the distribution pattern of GDF15 across different intraocular compartments in PDR and its potential biological implications.

Elevated intraocular GDF15 levels in PDR may reflect a local stress-related microenvironment associated with retinal ischemia, hypoxia, inflammation, and metabolic disturbance. Previous studies have shown that PDR is accompanied by increased angiogenic and inflammatory mediators, including VEGF and interleukins, which provides a plausible biological background for GDF15 upregulation (25, 26). However, because the present study only measured GDF15 concentrations in ocular fluids, it cannot determine the cellular source, signaling pathway, or functional role of GDF15 in PDR. Due to the low expression of GFRAL in peripheral tissues, GDF15 may act through GFRAL-independent pathways in the retinal microenvironment, and its specific receptors and downstream signaling pathways need further exploration (27, 28). Accordingly, any potential involvement of GDF15 in retinal inflammation, neurovascular stress, or tissue remodeling should be regarded as hypothesis-generating and requires further validation in tissue-based and mechanistic studies.

This study analyzed AH and VH samples from PDR patients obtained from independent cohorts. Although the comparison between AH and VH represents an exploratory inter-group analysis rather than an intra-individual paired comparison, the observed higher GDF15 levels in VH compared with AH remain biologically meaningful. This finding may suggest a potential compartment-related distribution pattern of GDF15 within the intraocular microenvironment of PDR, and is in line with the pathological characteristics dominated by ischemic injury in the posterior segment of the retina. However, this result should be interpreted as hypothesis-generating rather than mechanistic evidence. This trend provides useful preliminary evidence to support future well-designed paired-sample studies aimed at further elucidating the distribution pattern of GDF15 across different ocular compartments in PDR and its potential biological significance.

Several limitations related to baseline clinical characteristics should be acknowledged in this study. Due to the intraoperative collection of ocular fluid samples in clinical practice, complete baseline data were not available for all participants. In addition, the inherent difficulty in obtaining vitreous samples from non-diabetic surgical controls contributed to suboptimal age and sex matching between the PDR and control groups, which is particularly evident in the vitreous cohort and may introduce potential demographic confounding bias. Although exploratory correlation analyses were performed, the relatively small sample size and missing clinical information limited the statistical power and precluded reliable multivariable adjustment. Therefore, the potential influence of metabolic status, systemic vascular comorbidities, and prior ocular treatments on intraocular GDF15 levels cannot be fully excluded. Future prospective studies with larger sample sizes, standardized disease classification, better-matched control cohorts, and more complete clinical data collection are warranted to further validate the findings of the present study.

Given that current evidence regarding the cellular localization and mechanistic role of GDF15 in retinal tissues remains limited, further studies are warranted in the following directions. In subsequent cohort studies, we will strictly control for the effects of demographic imbalance and systemic comorbidities on intergroup variations in GDF15 concentrations. Prospective cohort studies including patients with different stages of DR could be established, with standardized protocols for simultaneous measurement of GDF15 in both ocular fluids and plasma. Meanwhile, multiplex analyses incorporating VEGF and inflammatory cytokines may help to evaluate the association between GDF15 and disease activity. Single-cell sequencing and tissue localization techniques could also be employed to clarify the cellular sources of GDF15 expression. Finally, *in vitro* and *in vivo* experimental models may be used to investigate the functional role and signaling pathways of GDF15. These future investigations may further clarify whether GDF15 can serve as a potential indicator for disease activity or prognosis in PDR, and may provide insights into its potential clinical relevance.

## 5 Conclusion

This study showed that GDF15 protein levels were significantly elevated in the AH and VH of patients with PDR compared with non-diabetic ophthalmic controls. These findings suggest that intraocular GDF15 upregulation is associated with PDR and may reflect local inflammatory, oxidative stress, or microvascular injury-related changes in the PDR microenvironment. Due to significant age and sex differences between groups, particularly in the vitreous cohort, as well as the potential influence of systemic metabolic and vascular comorbidities, these associations should be interpreted with caution. The cellular source, functional role, and clinical significance of GDF15 in PDR remain to be further clarified. Future studies with larger cohorts, standardized disease stratification, paired ocular fluid sampling, and mechanistic validation are needed to determine whether GDF15 has potential value in PDR disease assessment or therapeutic research.

## Data Availability

The original contributions presented in the study are included in the article/supplementary material. Further inquiries can be directed to the corresponding authors.
